# Tryptophan metabolism and plant health: mechanisms of action and application prospects

**DOI:** 10.3389/fpls.2026.1872762

**Published:** 2026-09-29

**Authors:** Xiaoting Li, Hua Bao, Xingwen Chen, Fangfang Zhou, Li Tang

**Affiliations:** 1 College of Resources and Environmental Science, Key Laboratory for Improving Quality and Productivity of Arable Land of Yunnan Province, Yunnan Agricultural University, Kunming, China; 2 Yunnan Vocational College of Agriculture, Kunming, Yunnan, China; 3 Yunnan Tobacco Company Qujing City Company, Qujing, Yunnan, China

**Keywords:** tryptophan, auxin, melatonin, plant health, crosstalk regulation, agricultural application

## Abstract

Pathogen infection poses a severe threat to the global crop yield and quality. Recent studies have highlighted the pivotal role of tryptophan and its metabolites in regulating plant disease resistance. However, owing to the highly branched nature of the tryptophan metabolic network and the functional pleiotropy of its products, the mechanisms by which these metabolites influence plant health have yet to be systematically elucidated. To address this gap, this review first summarizes the biosynthesis and metabolic routing of tryptophan in both plants and microorganisms, along with the key regulatory factors governing these processes. Building on this foundation, we elaborate on the multifaceted mechanisms through which tryptophan and its derivatives regulate plant health, including direct pathogen inhibition, reshaping of the soil microbiome, strengthening of cell wall physical barriers, crosstalk with reactive oxygen/nitrogen species (ROS/RNS), interactions with plant hormones, as well as induction of defense gene expression. Eventually, there is an in-depth discussion about the application prospects and major challenges of leveraging tryptophan metabolites in agricultural development. Altogether, our overarching goal is to establish a theoretical framework and chart new investigative avenues for fostering sustainable plant protection strategies and improving crop resilience.

**Systematic review registration:**

https://orcid.org/0009-0008-8550-2347.

## 1 Introduction

Tryptophan is an aromatic amino acid featuring an indole group. With the molecular formula C_11_H_12_N_2_O_2,_ its systematic chemical designation is α-amino-β-indole propionic acid. It primarily exists in the forms of L-type, D-type, and racemic DL-type isomers. As an essential amino acid for protein synthesis in all living organisms, it plays a fundamental role in the growth and development of plants (Arshad and Frankenberger, 1991). In plants, tryptophan serves as a central metabolic hub and is the precursor for a variety of structurally diverse downstream products. It participates in the biosynthesis of auxins (indole-3-acetic acid, IAA) and other indole compounds through indole-3-pyruvic acid (IPA) and indole-3-acetamide (IAM) pathways (Mano and Nemoto, 2012; Kasahara, 2016; Chen et al., 2018). Additionally, serotonin (5-hydroxytryptamine) and melatonin are generated by the tryptamine (TAM) pathway (Pelagio-Flores et al., 2011; Zhang et al., 2015), while indole glucosinolates (IGs) and camalexin are derived from the indole-3-acetaldoxime (IAOx) pathway (Glawischnig, 2007; Zhou et al., 2011; Sánchez-Pujante et al., 2017). In recent years, more studies have shown that the functions of tryptophan and its derivatives extend far beyond their primary metabolic roles as “metabolic precursors”, and are deeply involved in plant immune regulatory networks. Tryptophan-derived secondary metabolites (TDSMs) can not only directly exert toxicity against invading pathogens, but also act as signaling molecules in activating the downstream immune responses, constituting an important component of the innate immune system of plants (Wang et al., 2022a). For instance, indole has been reported to promote the defense of cotton against *Verticillium dahliae* (Miao et al., 2019). However, existing studies have largely focused on characterizing discrete metabolic pathways and specific functions of single tryptophan-derived compounds (Parthasarathy et al., 2018), rather than basal metabolism-mediated stress defense (Wang et al., 2025). However, currently, the research on the tryptophan metabolic network and how its structurally diverse products jointly regulate plant immune response through multiple synergistic mechanisms is still relatively limited. Hence, this review collected high-quality literature and databases in recent years, and analyzed the biosynthesis and transformation pathways of tryptophan and its metabolites in both plants and microorganisms. Based on the above, we summarized a regulatory framework for the mechanisms underlying plant health, with an emphasis on the network-level synergy among various pathways and their functional integration during plant-pathogen interactions. In addition, the agricultural application prospects and challenges are also discussed to provide a novel theoretical basis and control strategy for plant health management.

## 2 Biosynthesis and metabolic pathways of tryptophan

Tryptophan is mainly synthesized and metabolized by plants and microorganisms. Meanwhile, plants can also absorb and utilize tryptophan and its metabolites through root absorption or foliar spraying.

### 2.1 Tryptophan biosynthesis pathways

The subcellular localization of the tryptophan biosynthesis pathway differs markedly between plants and microorganisms (**Figure 1**). In plants, the study of the pathway dates to the late 1980s, with *Arabidopsis thaliana serving* as the main model (Last and Fink, 1988). Due to the fact that all proteins involved in tryptophan biosynthesis in *Arabidopsis* possess chloroplast transit peptide signal sequences at their N-termini, it is generally considered that tryptophan is primarily synthesized in chloroplasts (Singh and Matthews, 1994). Notably, recent research has shown that there is also a tryptophan biosynthesis pathway in the cytoplasm of higher plants. This pathway is catalyzed by the aminotransferase *VAS1* to synthesize tryptophan, phenylalanine, and tyrosine using phenylpyruvate, 4-hydroxyphenylpyruvate, and 3-indolepyruvate (Wu et al., 2024). In microorganisms, tryptophan biosynthesis was first characterized in the cytoplasm of *Escherichia coli*, and the classical tryptophan operon and attenuator models were proposed in molecular biology (Crawford, 1989). However, the core biochemical reactions are highly conserved between plants and microorganisms. The tryptophan biosynthetic pathway follows the main route of the shikimate pathway-chorismate-anthranilate-tryptophan. This evolutionary conservation suggests that it is essential for cellular physiology. The related pathway can be divided into three major stages (**Figure 1**). Firstly, the intermediate product of glucose metabolism from glycolysis and the pentose phosphate pathway is condensed to form 3-deoxy-α-arabinoheptulosonate-7-phosphate (DAHP). Subsequently, DAHP is converted into chorismate under the catalysis of a series of enzymes in the shikimate pathway. Eventually, glutamic acid and anthranilic acid are generated from chorismate and glutamine derived from the tricarboxylic acid cycle by anthranilate synthase (AS) (Mano and Nemoto, 2012), and the latter is sequentially catalyzed by transferase, isomerase, and tryptophan synthase (TS) to ultimately generate tryptophan (Berlyn et al., 1989; Zhao and Last, 1995). Among these processes, AS and TS are the key rate-limiting and regulatory enzymes (Niyogi and Fink, 1992; Li and Last, 1996). Notably, the tryptophan biosynthetic pathways of plants and microorganisms interact in natural environments, particularly in the rhizosphere. Plant tryptophan biosynthesis pathways can provide important nutrient sources for rhizosphere microbes through secretion. Conversely, indole compounds produced by microbial metabolism can regulate plant growth and metabolism through feedback. Tryptophan biosynthesis and its cross-kingdom interactions lay the foundation for the metabolic routing of tryptophan and the regulation of plant health.

**Figure 1:**
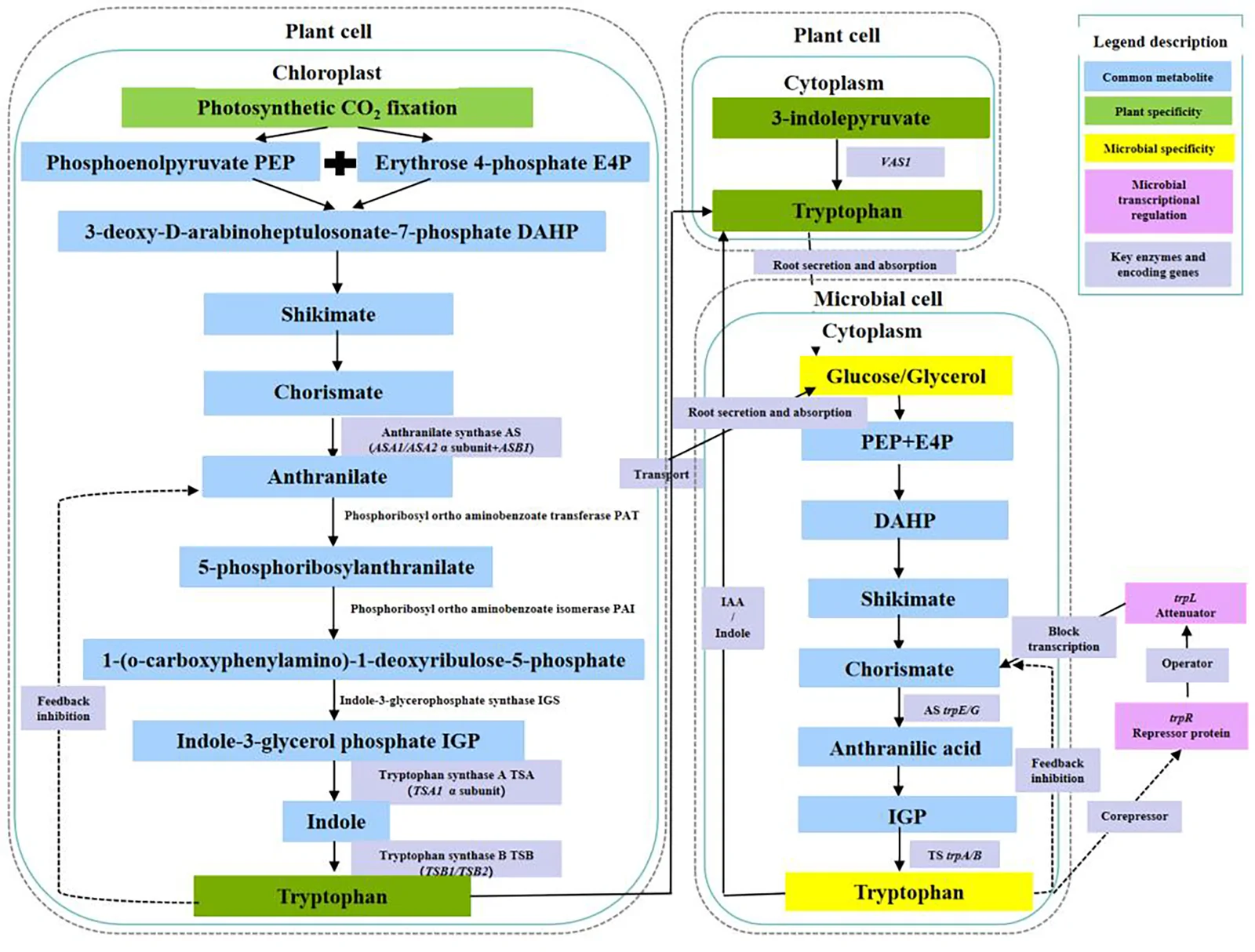
The biosynthetic pathway of tryptophan(compiled by Kriechbaumer et al., 2008; Galili et al., 2016; Wu et al., 2024).

### 2.2 Tryptophan metabolism pathways

Tryptophan metabolic pathways are known to encompass both anabolic and catabolic branches. On the one hand, tryptophan serves as a precursor for tissue protein that is related to the aromatic amino acid metabolism (Maéda and Dudareva, 2012; Lim et al., 2015), as well as lysine metabolic pathway (Yang et al., 2020). On the other hand, tryptophan catabolism generates a variety of products, including indole and pyruvate, which positively affect crop growth and development, alleviate stress, and enhance immunity (Watanabe and Snell, 1972).

In plants, tryptophan catabolism primarily flows to the biosynthesis of IAA, IGs and melatonin (**Figure 2**), among which IAA biosynthesis includes four distinct routes: IAOx pathway, IPA pathway, TMP pathway, and IAM pathway (Mano and Nemoto, 2012). The biosynthesis of IGs is another important branch of the IAOx pathway. In this route, indole-3-acetaldoxime serves as a critical branching point (Bak et al., 2001), which is synthesized under the action of CYP83B1 enzyme. It is further transformed into camalexin. These compounds are considered to play important roles in plant defense against pathogens (Pedras et al., 2002; Nongbri et al., 2012). Melatonin is a key metabolite of the TMP pathway (Wan et al., 2018). Accumulating evidence suggests that melatonin, its metabolites, and associated key enzymes contribute to plant resistance to adverse environments and reduce damage (Lee et al., 2015; Li et al., 2022a).

**Figure 2:**
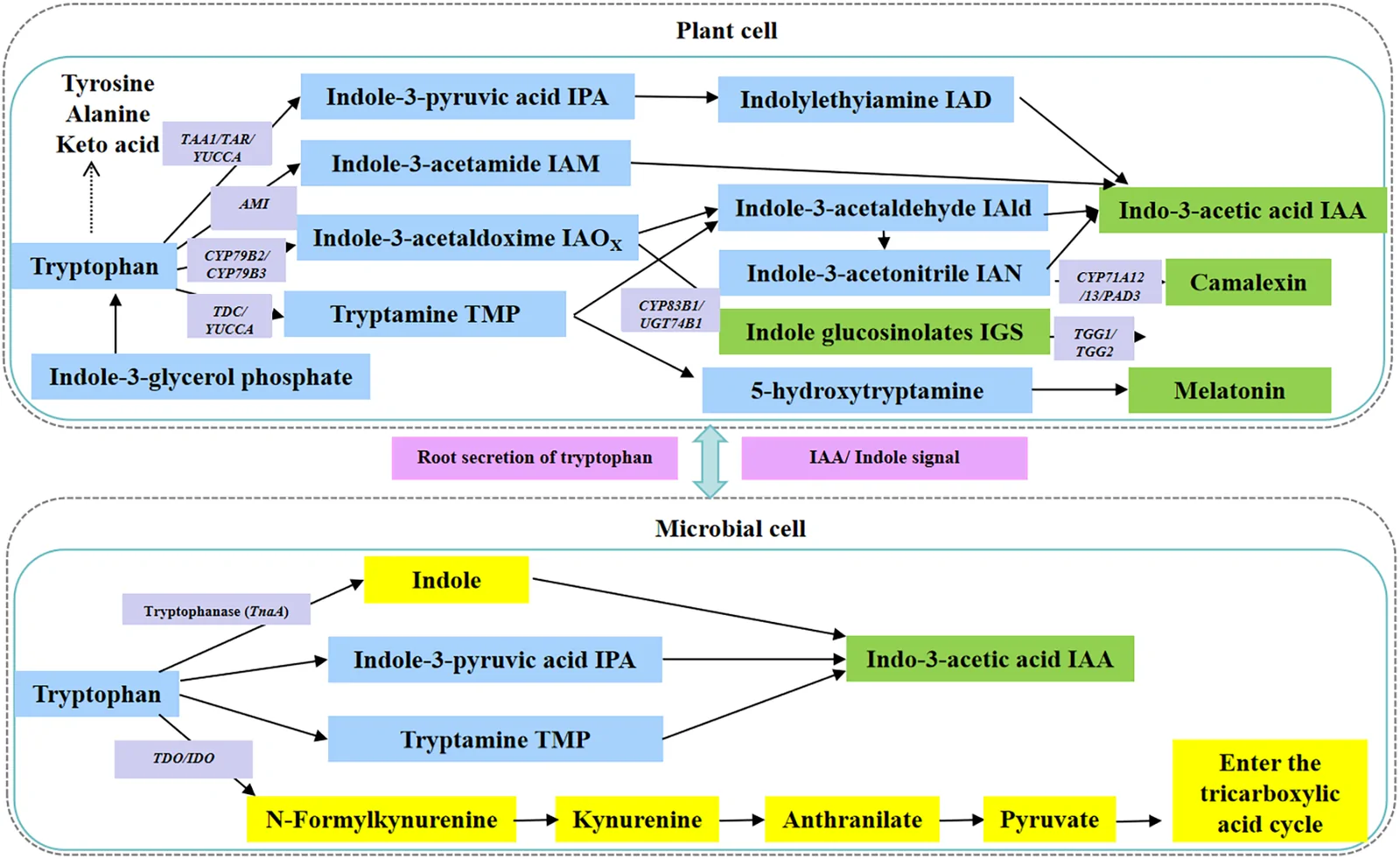
A conceptual diagram of tryptophan metabolism pathways (Modified from Wang et al., 2015; Wang et al., 2025).

In microorganisms, tryptophan catabolism proceeds through two major strategies, namely, the degradation of indole derivatives (including the indole, IPA, and TMP pathways) and small molecules, such as pyruvate (kynurenine pathway) (Zhang et al., 2019; Wu et al., 2021a). Importantly, tryptophan catabolism in plants and microorganisms does not proceed independently but forms a complex and dynamic interaction network mediated by tryptophan and its metabolites. Plant tryptophan metabolites have been reported to modulate the microbial behavior (Kulkarni et al., 2013; Shao et al., 2015; Zhang et al., 2021a), and microbial signaling molecules may regulate plant growth and immunity. This close association is essential for maintaining rhizosphere health. The tryptophan catabolism pathways in plants and microorganisms provide the material basis and mechanistic framework for studying the effects of tryptophan and its metabolites on plant immune response.

## 3 Factors influencing tryptophan biosynthesis and metabolism

An increasing number of studies have been shown that the biosynthesis of tryptophan and its metabolic pathways are influenced by multiple factors, which interact with each other to collectively determine the biosynthesis and related metabolic fluxes. Based on the nature of these influences, they can be classified into four aspects: growth regulation, genetic regulation, environmental regulation, and microbiome-mediated regulation.

### 3.1 Growth regulation

The contents of tryptophan and its metabolites have been reported to vary among species, tissue types, growth stages, and signaling interactions between species. For example, the tryptophan content in radish has been found to be approximately nine times higher than that in cucumber, sweet pepper, and tomato (Kamilova et al., 2006). Melatonin content in rice inflorescences is higher than that in leaves, both of which are thought to participate in regulating crop development and stress tolerance (Park et al., 2012). Camalexin and melatonin contents have been observed to change with growth stage and these changes are associated with disease resistance in potato, pepper, and eggplant (Shibata et al., 2010; Korkmaz et al., 2017; 2020). Interestingly, plants not only enhance resistance by modulating tryptophan and derivative levels, but also release indole that can affect neighboring individuals and induce the latter to emit volatile terpenes and other compounds, thereby enhancing the population defense against stresses, pests, and pathogens (Shen et al., 2018).

### 3.2 Genetic regulation

Genetic regulation of tryptophan biosynthesis and metabolism is mainly achieved through the expression and modification of key enzyme genes, the elimination of feedback inhibition of metabolic pathways, and the interplay with related amino acid metabolic routes. AS (encoded by *trpE*) is recognized as a rate-limiting enzyme for tryptophan biosynthesis, and overexpression of its anti-feedback inhibition mutant gene *trpEfbr* has been shown to significantly increase tryptophan production (Liu et al., 2018). Tryptophanase (TnaA) catalyzes the decomposition of tryptophan into indole and L-serine, and knockout of the *TnaA* gene has been observed to reduce tryptophan degradation and promote its accumulation *in vivo* (Liu et al., 2012). Meanwhile, tryptophan biosynthesis is inhibited by the feedback of phenylalanine and tyrosine, and blocking the biosynthesis routes of the latter two may help increase the yield of tryptophan (Liu et al., 2016). Increasing the supply of tryptophan metabolic precursors and promoting the metabolic flux may also promote overall metabolic output. For instance, enhancing the supply of glucose carbon, promoting the flux into the pentose phosphate pathway (PPP), elevating the erythrose-4-phosphate (E4P) ratio can increase metabolic output (Long et al., 2017). In addition, elevated tryptophan levels have been associated with high lysine in plants. The accumulation of lysine has been found to induce tryptophan biosynthesis and metabolism in transgenic rice with high free-lysine content (Yang et al., 2020).

### 3.3 Environmental regulation

Environmental regulation of tryptophan metabolism may be considered from two aspects: abiotic and biotic factors. Abiotic factors include light, mechanical damage, and soil physical and chemical properties. Biotic factors mainly refer to stresses such as pathogen infection. Among abiotic factors, the contents of tryptophan and melatonin in plants have been reported to be higher during the light period (Korkmaz et al., 2017), and they are also subject to degradation by ROS (Bellmaine et al., 2020). Mechanical wounding (Zeng et al., 2016; Asai et al., 2017) and herbivore feeding (Erb et al., 2015) have been shown to induce the expression of biosynthetic enzyme genes such as *TSA* and *TSB*, promote the release of indole, and upregulate the metabolism of nicotinic acid and nicotinamide, thereby activating plant growth and defense responses. Electric field driving has also been reported to affect tryptophan metabolism and trigger related defense mechanisms (Zhang et al., 2023). In addition, the contents of tryptophan metabolites are significantly different among soil types. Soil pH, organic carbon content, and cation exchange capacity (CEC) may influence IAA levels (Sarwar et al., 1992), and soil humic acids may also affect tryptophan decomposition (Chalvignac and Mayaudon, 1971). Among biotic factors, changes in plant root exudates may cause soil microbial aggregation, which in turn regulates tryptophan metabolism, recruits specific beneficial bacteria, and enhances disease resistance during pathogen infection (Du et al., 2022; Zhou et al., 2023).

### 3.4 Microbiome-mediated regulation

Soil microorganisms are considered to be crucial in the absorption, transformation, and metabolic regulation of tryptophan, and exert a profound impact on the growth and health of plants (Sarwar et al., 1992; Walitang et al., 2017), which are regarded as an important part of maintaining healthy soil ecological functions (Nakano et al., 2016; Nakazaki et al., 2019; Johnson, 2019; Du et al., 2022; Zhou et al., 2023). Microorganism-mediated regulation may mainly be reflected in microbial gene resources, microbial metabolic regulation, microbial community homeostasis, and endoplasmic reticulum (ER) processes. Soil microorganisms have been found to carry multiple tryptophan metabolism-related genes, such as *trpS*, *trpB*, *trpH*, *trpR*, and *trpA*, that are involved in tryptophan metabolism and regulate plant growth and disease prevention (Ma et al., 2023a; Zhang et al., 2022a). Meanwhile, microorganisms in the soil have been reported to modulate rhizosphere tryptophan metabolism and utilize tryptophan and other small-molecule substances exuded by plant roots to synthesize and release IAA, promoting the growth and health of plants (Yuan et al., 2013; Shao et al., 2015; Liu et al., 2017; Walitang et al., 2017; Wang et al., 2021; Zhang et al., 2022a; Figueredo et al., 2023). In addition, there are also multiple tryptophan metabolic pathways in microorganisms (Liu et al., 2019). Microbial communities associated with *Arabidopsis thaliana* roots has been reported to regulate crop growth and development through interactions that convert tryptophan to IAA (Lu et al., 2018). Furthermore, rhizosphere bacterial symbionts and plant tryptophan metabolism jointly mediate microbial community homeostasis and health (Wolinska et al., 2021). Studies have reported that the endoplasmic reticulum contains abundant PYK10 myrosinase, which is capable of hydrolyzing tryptophan-derived IGs, modulating tryptophan metabolism in plant root exudates, and promoting or inhibiting the growth of certain microorganisms to reduce disease incidence (Nakano et al., 2016; Nakazaki et al., 2019; Johnson, 2019).

## 4 Functions and mechanisms of tryptophan in regulating plant health

According to the analysis of tryptophan biosynthetic and metabolic pathways and influencing factors, in plants and microorganisms, tryptophan is not only an essential amino acid for protein synthesis, but is also transformed into a series of metabolites with different functions. Together, they form a highly multifunctional metabolic hub in the innate immune system of plants. Therefore, the mechanisms of tryptophan and its metabolites inhibiting pathogens may be summarized as follows.

### 4.1 Mechanisms of tryptophan and its metabolites in pathogen inhibition

#### 4.1.1 Tryptophan accumulation as a broad-spectrum defense signal for pathogen inhibition

The accumulation of tryptophan itself has been found to trigger plant defense responses. Liu et al. (2010) reported that the mutations of the cytochrome P450 protein CYP82C2 led to substantial accumulation of tryptophan, thus regulating the broad-spectrum defense against various pathogens. demonstrated that companion planting altered the composition of tomato root exudates, increasing the secretion of tryptophan and effectively reducing the incidence of spotted wilt. These findings suggest that changes in tryptophan concentration may not only be perceived as “metabolic signals” of plant immune response, but also recruit beneficial microbes, potentially fulfilling dual functions of recruitment and inhibition and suggesting the presence of an unidentified “tryptophan sensor” for future investigation.

#### 4.1.2 Tryptophan metabolites for pathogen inhibition

Tryptophan metabolites such as IAA, serotonin, and melatonin have all been reported to exert regulatory effects on plant immune response (Hiruma et al., 2013; Frerigmann et al., 2016; Pastorczyk et al., 2019; Kosaka et al., 2021). IAA is the core product of the tryptophan metabolic pathway. Navarro et al. (2006) first reported the relationship between IAA and the ability to resist pathogens. Wang et al. (2024) further confirmed that overexpression of the auxin-responsive gene *MdIAA24* could significantly improve defense against *Colletotrichum* spp. infection. Interestingly, IAA appears to play a dual role in plant defense. Optimal IAA concentrations enhance resistance by reinforcing cell wall integrity and inducing pathogenesis-related (*PR*) gene expression to enhance resistance (Majda and Robert, 2018; Liu et al., 2024). Conversely, elevated IAA levels promote susceptibility through the activation of salicylic acid (SA) signaling (Li et al., 2023b). This indicates that the IAA-mediated defense effect may be related to multiple mechanisms including cell wall metabolism, plant hormone signaling, and defense gene expression in addition to direct inhibition.

Serotonin is an important tryptophan-derived metabolite. It has been shown to exhibit direct defense activity against *Fusarium graminearum* (Pasquet et al., 2014). Particularly following *Bipolaris sorokiniana* infection, serotonin accumulation is rapidly induced in response to pathogen attack (Ishihara et al., 2017). In addition, serotonin is thought to possess dual functions of “structural repair” and “chemical inhibition.” Ishihara et al. (2008) revealed that the increased expression of the AS gene significantly elevates serotonin content in diseased tissues to repair browning lesions and inhibit the expansion of fungal hyphae. Meanwhile, Lu et al. (2018) reported that there is a functional coupling between serotonin defense and SA signaling. *CYP71A1* gene increases SA content and decreases serotonin levels through transcriptional regulation, which appears to determine the resistance or susceptibility of rice to pests.

Melatonin is one of the most diverse members of the tryptophan metabolic network. On the one hand, it may directly interfere with pathogen virulence gene expression. After plants are treated with melatonin, the expression of virulence-related genes is significantly down-regulated in *Phytophthora nicotianae* (Zeng et al., 2022). On the other hand, melatonin can activate defense pathways involving its biosynthesis genes. After common mosaic virus (CsCMV) infection, the transcription of melatonin biosynthesis genes (*MeTDC2*, *MeT5H*, *MeASMT1*) and catalase genes (*MeCAT6*, *MeCAT7*) has been observed to be activated and to positively regulate resistance to CsCMV (Wei et al., 2023). Melatonin exhibits multi-target suppress pathogen and virulence gene inhibition.

IGs and camalexin derived from tryptophan metabolites play a crucial role in plant immune response (Soledade et al., 1997; Nguyen et al., 2022; Liao et al., 2022). Upon pathogen infection, IGs are hydrolyzed by myrosinase to release compounds such as thiocyanates, nitriles, and isothiocyanates. These decompositions exhibit broad-spectrum defensive activity against pathogens (Chen et al., 2020; Coca-Ruiz et al., 2024). IGs may also regulate plant disease and insect resistance through crosstalk with IAA homeostasis (Malka and Cheng, 2017; Ye et al., 2019). These results reflect the deep integration of “defense output” and “hormonal regulation” in the tryptophan metabolic network.

Camalexin is also known as the main product derived from tryptophan, which is reported to directly inhibit the growth of *Verticillium* spp. *in vitro* (Iven et al., 2012) and may also trigger systemic induced resistance (ISR) against *Botrytis cinerea*, *Pseudomonas syringae* pv. *tomato* DC3000, and *Phytophthora capsici* (Li et al., 2023a). Furthermore, increased camalexin secretion may promote the colonization of the beneficial bacterium CH267, which is advantageous for crop growth (Kopřivová et al., 2023). This observation suggests that camalexin may not only exert direct antimicrobial effects but also indirectly enhance plant defense through remodeling of the rhizosphere microbial community.

Other tryptophan metabolites also have different effects on pathogens. Luo et al. (2016) revealed that IAA and TAM have a slight positive effect on the production of the mycotoxin 15-ADON, whereas indole-3-acetonitrile (IAN) significantly inhibits its production. Zhu et al. (2018) demonstrated that the secondary tryptophan metabolites, including phenylacetaldehyde, indole-3-acetamide, daidzein, hypoxanthine, and octanal, are involved in defense responses to *Phytophthora sojae*. These findings show that different branch products in the tryptophan metabolic network have distinct regulatory effects on pathogens. Thus, pathogen infection may be inhibited by modulating metabolic flux in plant disease management and variety-specific applications.

#### 4.1.3 Transcription factors, enzymes, and transporters for pathogen inhibition

Different branches of the tryptophan pathway are selectively activated to prevent pathogen infection. Transcription factors, such as MYB34, MYB51, and MYB122, have been reported to promote resistance to *Podosphaera cucumerina* by regulating IGs biosynthesis (Frerigmann et al., 2016). PEN2 (a leaf-localized myrosinase) has also been reported to activate IGs in response to pathogen invasion (Frerigmann et al., 2016). WRKY33 rapidly responds to pathogen infection and induces IGs biosynthesis and specific side chain modification pathways (Tao et al., 2022). Moreover, the activation of CYP81F2 and PEN2 increases camalexin accumulation in infected roots and enhances plant resistance (Iven et al., 2012). The transporter AtABCG34 is responsible for secreting camalexin to the leaf surface to help prevent *Alternaria brassicicola* infection (Khare et al., 2017). Collectively, these findings suggest that transcriptional regulation of tryptophan biosynthesis and metabolic processes also represents a key to inhibiting pathogens.

Tryptophan and its derived metabolites exert multifaceted antimicrobial functions. Beyond their direct pathogen-killing effects, tryptophan and its metabolites may potentiate host resistance by activating the immune signaling cascades, reinforcing cell wall architecture, orchestrating phytohormone crosstalk, inducing defense gene expressions, as well as selectively recruiting beneficial rhizosphere microorganisms. Nevertheless, the efficacy of these metabolites is not uniform, but are intrinsically governed by pathogen species, dosage, and the local biochemical microenvironment. Looking to the future, key research directions include clarifying the dynamics of metabolic flux, the developing high-resolution *in situ* quantitative methods, and characterizing the metabolic transporter systems. Filling these gaps will not only help deepen our understandings of the tryptophan-mediated immune mechanisms but also guide the rational design of agricultural intervention measures based on metabolites.

### 4.2 Mechanisms of pathogen inhibition through interactions between tryptophan and its metabolites with soil microorganisms

As the second genome of plants, the rhizosphere microbiome functions through signal and metabolite interactions among microbial members to maintain plant health, but also operates via plant–microbiome crosstalk (Finkel et al., 2019; Harbort et al., 2020). Plants are known to secrete a variety of metabolites to shape the rhizosphere microbial community, and these metabolite–microorganism interactions in turn influence plant health status to a large extent (Riseh et al., 2024b; Riseh et al., 2024a). Early studies have found that tryptophan content is the highest in the rhizosphere soil region 12 to 16 cm from the root tip, which first confirmed the presence of tryptophan in the rhizosphere soil (Jaeger et al., 1999). Since then, research on the interaction of tryptophan and its metabolic pathways with soil microorganisms in defense against diseases have been successively reported.

#### 4.2.1 Secretion of plant tryptophan and its metabolites: A dual genetic-pathogen cue for beneficial microbiome recruitment

Plant root exudates are considered an important source of tryptophan and its metabolites in the rhizosphere. For example, differences in tryptophan metabolism among rice varieties in the rhizosphere regulate the root-associated microbiota, thus affecting resistance to the rice blast fungus (Tian et al., 2021). In addition, pathogen infections such as *Phytophthora sojae* and *Fusarium culmorum* have been observed to induce the accumulation of tryptophan or its metabolites in plant roots and recruit beneficial microorganisms to defend against pathogens (Stasko et al., 2020; Piasecka et al., 2022).

#### 4.2.2 Microbial tryptophan transformation to IAA: A conserved signaling nexus between synthetic consortia and plant immune response

The tryptophan secreted by plants might be utilized by the rhizosphere microorganisms, including *Pseudomonas fluorescens*, *Bacillus* spp., *Stenotrophomonas maltophilia*, *Streptomyces* spp., and the fungus *Metarhizium robertsii*, which have been shown to enhance plant resistance by transforming tryptophan and its metabolites when plants are challenged by pathogens (Weston et al., 2012; Balah et al., 2018; Singh and Jha, 2017; Worsley et al., 2020; Liao et al., 2017; Maier et al., 2021). Furthermore, synthetic microbial communities have also been reported to possess similar defense functions (Maier et al., 2021). The most typical example is IAA. IAA acts as an important signaling molecule in plant–microbe interactions, and IAA signaling has been suggested to be an important component of plant defense against pathogens (Spaepen and Vanderleyden, 2010).

#### 4.2.3 Inhibition of pathogens through interactions of tryptophan and its metabolites with microorganisms

Wolinska et al (2021) pointed out that the plant’s endogenous tryptophan immune system may be insufficient to completely protect plants from fungal infection, while the rhizosphere bacterial partners appear to provide an additional layer. This observation suggests that in addition to directly participating in defense, a more important role of tryptophan and its derivatives released by plants is to indirectly inhibit pathogens by selectively recruiting beneficial microorganisms. This process is one of the core mechanisms of microbiome-mediated disease suppression. Increased tryptophan content has been found to facilitate the colonization of *Bacillus amyloliquefaciens* and to reduce the occurrence of disease (Liu et al., 2017). IAA improves the relative abundance of phyllosphere microorganisms, especially fungal functional groups involved in the regulation of plant pathogens (Shao et al., 2024). IGs have also been shown to shape rhizosphere bacterial community structure and increase the relative abundance of rhizosphere core OTUs (Chroston et al., 2024). Myrosinase modulates the tryptophan metabolism secreted by roots, and then influences the assembly of root microbiota, which may have important implications for plant–microbe interactions (Basak et al., 2023). In addition, benzoxazinoids of tryptophan-derived secondary metabolites have been reported to affect plant growth and health by regulating the correlations between root metabolites and rhizosphere bacterial and fungal communities (Cotton et al., 2019). Collectively, there are positive synergistic interactions between tryptophan and its metabolites and root-associated microbiota in the defense against pathogen infection (Kudjordjie et al., 2022).

Tryptophan and its metabolites orchestrate plant–soil microbiota interactions through multilayered mechanisms, encompassing root secretion, microbial biotransformation, and the selective recruitment of beneficial consortia. Critically, this metabolic crosstalk is not a unidirectional flux but rather a dynamic reciprocal feedback network. However, our current understanding of this feedback remains fragmentary, particularly regarding the spatiotemporal dynamics of tryptophan-derived signals across different growth phases, spatial root compartments, and concentration gradients, as well as their impact on microbial community composition, structure, and functional redundancy. Conversely, the precise feedback effects of microbial tryptophan derivatives and associated signaling molecules on host growth, development, and immunity demand deeper investigation, especially under ecologically complex and fluctuating field conditions. In this context, melatonin has recently emerged as a pivotal regulator that enhances crop stress tolerance by actively reshaping rhizosphere microbiome architecture (Ye et al., 2022; Cao et al., 2024). Looking forward, our research will prioritize the systems-level dissection of dynamic interaction networks linking tryptophan metabolism to microbiome assembly, coupled with predictive modeling for integrated disease management. These efforts aim to establish novel theoretical paradigms and translational strategies for sustainable plant health protection.

### 4.3 Mechanisms by which tryptophan and its metabolites regulate plant health via the cell wall

The cell wall is the first physical barrier in plant defense against pathogen invasion. When attacked by pathogens, plants are able to enhance resistance through rapid and precise cell wall remodeling. Studies have shown that cell wall attachments are deposited as structural polymers such as callose, lignin, and suberin, forming physical barriers, reinforcing cell wall resistance, protecting host cells from degradation by pathogen-secreted enzymes, and preventing pathogens from invading plant tissues and spreading (Underwood, 2012; Wang et al., 2023; Munzert and Engelsdorf, 2024). In this process, tryptophan and its metabolites may regulate plant health either by affecting the degradation activity of pathogen cell walls or by enhancing the function of plant cell walls.

#### 4.3.1 Effects of tryptophan and its metabolites on pathogen cell wall degradation activity

Ohashi and Chaleckis (2021) revealed that tryptophan accumulation reduces the stress tolerance of pathogens to cell wall or cell membrane damage through the cell wall integrity (CWI) pathway. In other words, tryptophan may interfere with the signal transduction processes of pathogens in response to cell wall stress, thereby potentially inhibiting microbial infection capability. Although tryptophan metabolites have been reported to possess broad-spectrum antimicrobial activity, whether they achieve inhibition of infection by affecting the biosynthesis or degradation activity of pathogen cell wall remains to be further investigated.

#### 4.3.2 Effects of tryptophan and its metabolites on enhancing cell wall structural function

Studies have suggested that activation of the tryptophan metabolic pathway is an important component of cell wall-enhanced defense responses. Song et al. (2024) compared resistant and susceptible rose varieties following *Marssonina rosae* infection and found significant synergistic changes between cell wall defense-related biological processes and tryptophan metabolism in resistant varieties. After wheat infection by *Fusarium* (Li L. et al., 2023) and lemon infection by oil spot (Zhou et al., 2021), the expression of tryptophan biosynthesis genes is significantly upregulated, which strengthens the structural integrity of the primary cell wall and enhances lignin synthesis pathways, promoting the accumulation of lignin and preventing the invasion of pathogens. IAA plays a direct regulatory role in cell wall synthesis and deposition (Majda and Robert, 2018). Zhou et al. (2023) confirmed that exogenous IAA may enhance cell wall resistance to pathogen invasion and affect fruit quality by regulating the expression of cell wall metabolism-related genes such as *PG* (polygalacturonase), *PME31* (pectin methylesterase 31), and *PME63*. This observation indicates that IAA exerts dual functions in cell wall defense and developmental regulation. IGs and myrosinase constitute the classic chemical defense systems of Cruciferous plants. In recent years, the association between cell wall physical defense and these compounds has been gradually revealed. Clay (2011) showed that flg22 (a conserved peptide of bacterial flagellin) induces the metabolism of IGs in *Arabidopsis*, which is closely related to callose accumulation, and the decomposition products of IGs serve as upstream signaling molecules for callose deposition. Furthermore, Ahuja et al. (2021) reported that the absence of myrosinase not only altered the endogenous chemical composition, but also affected the plant physical structure and the integrity of physical defense barriers, indicating that myrosinase is of importance in plant physical defense. Specifically, Zhang et al. (2019) reported that the myrosinase gene *TGG1* plays a key role in stomatal defense, preventing pathogen invasion by regulating stomatal closure. Melatonin demonstrates remarkable versatility in enhancing cell walls. Studies have shown that it increases the lignin content and cell wall thickness to enhance cell wall strength (Zhao et al., 2022), enhances the resistance of harvested fruits to anthracnose disease (Lu et al., 2024), and delays fruit softening and cold injury (Li et al., 2024; Bhardwaj et al., 2022), demonstrating its dual application potential in cell wall defense and agricultural quality regulation.

To date, the role of tryptophan metabolites in cell wall-mediated immunity remains largely unexplored. Key unanswered questions include the genetic regulation of cell wall-specific defenses, the associated structural dynamics, the cross-kingdom functionality of IGs beyond Brassicaceae, and the interplay among multiple defense modules - all of which are constrained by the fundamental trade-off relationship between growth and immunity. Clarifying these networks is of great significance for advancing our understanding of pathogen resistance from a trade-off limitation to a functional synergy.

### 4.4 Mechanisms of regulating plant health by tryptophan metabolites as redox rheostats: integrating direct ROS/RNS scavenging, IAA-mediated feedback, and melatonin-orchestrated homeostasis

ROS and RNS are key regulatory factors in plant life activities with dual identities as “signaling molecules” and “toxic factors” (Thiruvengadam et al., 2024). Their interplay generates defensive responses to pathogen attack (Río, 2015). ROS/RNS bursts induce lipid peroxidation, activate protein kinases, and regulate defense gene expression, in turn leading to local programmed cell death (hypersensitive response HR) and resistance to pathogens (Hancock et al., 2002; Iqbal et al., 2023; Park and Son, 2024; Khan et al., 2023). However, excessive accumulation of ROS/RNS also causes oxidative damage, therefore, their production and elimination might be subject to precise homeostatic regulation. Tryptophan and its metabolites play three roles in this regulatory network: direct scavengers, inducers, and homeostatic regulators. These functions are not mutually exclusive but are dynamically switched based on the type of metabolite, concentration, cellular environment, and stress type. Hence, elucidating the interaction modes between tryptophan metabolites and ROS/RNS is crucial for understanding the redox dimension of plant immune regulation.

#### 4.4.1 Integration of tryptophan with ROS/RNS for pathogen inhibition

It was found that the algicidal mechanism of tryptophan against *Pseudomonas aeruginosa* may be attributed to excessive ROS production under light conditions (Wu et al., 2022b). In addition, as an upstream environmental signal, appropriate light alters the pattern of ROS output by regulating tryptophan metabolic flux, affecting the establishment of plant defense response (Shimizu et al., 2021). Meanwhile, pathogen infection enhances the expression of genes related to tryptophan metabolism, plant hormone biosynthesis and ROS homeostasis, and activates multiple pathways synergistically to collectively strengthen plant immune response (Chen et al., 2023). These observations suggest that the integration of tryptophan metabolism with ROS homeostasis is not limited to direct chemical interactions between metabolites and free radicals. It can also occur at the level of transcriptional regulation, or through the integration of multiple mechanisms to improve plant health.

#### 4.4.2 Feedback interaction between IAA and ROS in pathogen inhibition

IAA itself regulates the production of ROS. Tzipilevich et al. (2021) revealed that IAA secreted by bacteria acts on plant immune response and ROS toxicity, and ROS feedback induces IAA and promotes bacterial rhizosphere colonization. This dual regulation may enhance plant protection against fungal infection and indicates that there is a positive feedback loop between IAA and ROS. Moreover, carbonylated proteins (RCS) are identified as key factors in signal transduction between IAA and ROS (Biswas et al., 2019), revealing the bridging role of oxidative modification in IAA signal transduction. Besides, crosstalk of IAA and ROS regulates meristem activity to adapt to environmental stress (Tognetti et al., 2017), suggesting that IAA and ROS interaction may represent the core mechanism of plant developmental plasticity and stress adaptation. In addition, the overexpression of tryptophan aminotransferase gene *AtYUC6 regulates* ROS homeostasis and enhance drought tolerance during IAA biosynthesis in potato (Park et al., 2013), which demonstrates that tryptophan metabolism may play important roles in abiotic stress adaptation involving ROS.

#### 4.4.3 Melatonin-ROS/RNS interaction in pathogen inhibition

Melatonin interacts with ROS/RNS and participates in a variety of physiological responses through three functions of direct scavenging activity, trigger of induced defense, and steady-state maintenance regulation (Pardo-Hernández et al., 2020). It has been found that melatonin can directly scavenge hydroxyl radicals (·OH), peroxyl radicals (ROO·), peroxynitrite anions (ONOO^8^), and singlet oxygen (¹O_2_), and its scavenging efficiency is higher than that of classical antioxidants (Reiter et al., 1999). Moreover, melatonin induces a series of immune responses to ROS and RNS bursts in plants, and induces the biosynthesis of H_2_O_2_ (Ali et al., 2020). Furthermore, the core function of melatonin is not simply to “eliminate” ROS, but to maintain H_2_O_2_ at homeostatic levels, maintaining it neither below the signaling threshold (avoiding immune insensitivity) nor above the damage threshold (avoiding cellular toxicity). This is mainly reflected in the ability of melatonin to decrease H_2_O_2_ biosynthesis and maintains it at a steady state level, thus producing resistance (Ali et al., 2020). In addition, melatonin acts as a regulator of the mitogen-activated protein kinase (MAPK) cascade and ROS/RNS feedforward loop during the plant-pathogen interaction (Mansoor et al., 2024), and it is essential for maintaining the balance between plant disease resistance and development. This “homeostatic regulation” reflects the essence of melatonin as a signal integrator.

#### 4.4.4 Interaction of other tryptophan metabolites with ROS/RNS in pathogen inhibition

Tryptophan derivatives such as pinoline, N-acetylserotonin, tryptamine, N-phthaloyltryptamine, and N-prenyltryptamine have free radical scavenging activity, among which N-alkylated tryptophan exhibits the highest scavenging activity against ROS and RNS (Reiter et al., 1999; Estevão et al., 2010). These results suggest that the N-position modification on the tryptophan skeleton may significantly enhance its antioxidant capacity, providing chemical clues for the design of more efficient antioxidant strategies.

The interaction of tryptophan and its metabolites with ROS/RNS constitutes a dual-layer regulatory system involving both direct chemical action and signal-network integration in plant immune regulation. The crosstalk among auxin, ROS, and nitric oxide (NO) is involved in plant development and abiotic stress (Parveen et al., 2022). However, whether the crosstalk between other tryptophan metabolites and ROS/RNS affects plant immune response, and how they are regulated by transcription factors remain to be systematically investigated. Meanwhile, the *Bacillus* spp. perhaps triggers the outbreak of ROS in plant leaves and roots, and the ROS tolerance of *Bacillus* is higher than that of pathogens, thus promoting *Bacillus* colonization and resistance against pathogen invasion (Zhang et al., 2021b). The contribution of tryptophan metabolites to this selective ROS tolerance difference is an open question in current research. Furthermore, the transcription factor WRKY55 in the tryptophan metabolic pathway regulates the synthesis and homeostatic balance of ROS and SA, affecting plant growth, development, and resistance to pathogens (Krishna, 2022; Wang et al., 2020). These findings indicate that tryptophan and its metabolites crosstalk with ROS/RNS and plant hormones to form multiple systemic defense mechanisms. Therefore, the interaction of tryptophan and its metabolites and plant hormones will be discussed in depth.

### 4.5 Mechanisms by which interactions between tryptophan, its metabolites, and plant hormones regulate plant health

The crosstalk among plant hormones, including SA, jasmonic acid (JA), ethylene (ET), abscisic acid (ABA), gibberellins (GA), cytokinins (CK), and IAA forms a highly dynamic, context-dependent signaling system. Studies have shown that the production and signal response of plant hormones significantly change during pathogen infection (López et al., 2008; Zhao and Li, 2021). For example, the multiple hormone pathways such as SA, JA, ABA, IAA, and CK, are simultaneously activated after soybean mosaic virus (SMV) infection (Han et al., 2023), and PR gene expression is possibly jointly regulated by IAA, CK, JA, and SA (Wei et al., 2021a; Han et al., 2022). Tryptophan and its metabolites possibly act as “regulatory knobs” in the network to regulate plant defense by influencing hormone synthesis, signal transduction or responsive gene expression.

#### 4.5.1 Crosstalk between tryptophan and its metabolites with plant hormones in pathogen inhibition

IAA does not function in isolation, but is interconnected with plant hormones, forming a complex regulatory network to govern plant growth and defense (Kazan and Manners, 2009; Naseem et al., 2015; Ma et al., 2023b). Among them, the relationship between IAA and the defense hormones SA and JA is the most intensively studied but also the most controversial field. IAA produced by pathogens promotes their own growth. In contrast, high concentrations of IAA inhibit plant growth and promote susceptibility by suppressing SA-mediated defense (Sarwar and Kremer, 1995; Kidd et al., 2011; Djami-Tchatchou et al., 2020). IAA also serves as a microbial signal molecule to regulate the expression of bacterial virulence genes (McClerklin et al., 2018). However, IAA may also directly enhance resistance through SA and JA (Zhang et al., 2007; Ding et al., 2008; Coelho et al., 2019; Wei et al., 2021b), while *CAT2* coordinates the biosynthesis of SA, IAA, and JA (Yuan et al., 2017). These observations not only suggest that the immune effect of IAA has a strict concentration window, but also indicate that IAA cooperates with SA and JA to reduce disease occurrence (Li et al., 2023b). Furthermore, the coordination of IAA and JA requires the initiation of acyl-homoserine lactone (AHL), which may provide a new strategy for the prevention and control of crop diseases (Liu et al., 2022a). Indole released by plants not only serves as a volatile signal to affect neighboring plants, but also activates the transcription of *OsWRKY70* and JA biosynthesis genes, and enhances JA signaling transduction to reduce the damage of phytophagous insects (Erb et al., 2015). These results provide direct evidence that tryptophan metabolites can regulate biotic stress response through the JA pathway.

Melatonin is considered a key molecule in plant immune responses (Arnao and Hernández-Ruíz, 2017). It is characterized by the ability to interact simultaneously with multiple hormone pathways. Melatonin (10 μM) induces the expression of a series of defense genes activated by SA and ET (Lee et al., 2014). The crosstalk between melatonin and GA activates terpenoid synthesis and plant hormone signal transduction pathways, affecting plant–pathogen interactions (Di et al., 2019). In addition, TDSMs also interact with the SA defense system to maintain plant homeostasis and defend against pathogen invasion (Wang et al., 2022a).

#### 4.5.2 Direct connection between tryptophan and its metabolites with plant hormones in pathogen inhibition

Tryptophan synthase β-subunit 1 (TSB1) has been found to coordinate plant growth and abiotic stress responses by balancing tryptophan and ABA homeostasis (Liu et al., 2022b). This finding reveals the direct metabolic–hormonal junction between tryptophan metabolism and ABA signaling, transcending the traditional crosstalk level of signaling pathways, and offers new research perspectives for plant immune regulation.

#### 4.5.3 Accumulation of tryptophan and its metabolites with plant hormone-induced substances in pathogen inhibition

There is a positive synergistic relationship between tryptophan-derived IGs and SA defense system. IGs mediate programmed cell death via genes activated by the SA pathway and improve plant immune response (Fukunaga et al., 2017). Although SA-mediated signal transduction may weaken the immunity triggered by flg22, it induces the biosynthesis and accumulation of IGs and enhances the resistance to *Penicillium brasiliense* (Yi et al., 2022). Furthermore, knockout of the tryptophan synthase gene *GbTSA1* has been reported to cause cell death and high accumulation of indole and indole metabolites, which may enhance cotton disease resistance (Miao et al., 2019). JA treatment has been shown to increase IG content and endow crops with resistance to gray mold and *Alternaria* leaf blight (Liu et al., 2010; Choudhury et al., 2022). The application of both SA and methyl jasmonate (MeJA) induces the systemic accumulation of IGs and reduce disease incidence (Zang et al., 2015).

The interactions of tryptophan and its metabolites with plant hormones constitute an immune regulatory network, including crosstalk among IAA, melatonin, and other plant hormones mediated by transcription factors or signaling components; tryptophan-ABA homeostasis; and plant hormone-induced IG accumulation. However, IAA and ABA jointly participate in *Pseudomonas fluorescens-mediated scab control in barley*- (Petti et al., 2012). Future challenges lie in analyzing the multiple mechanisms mediated by rhizosphere microbial transformation in the complex plant–hormone–microorganism tripartite interactions, identifying the nodes and metabolites that determine key control points, and deepening research on tryptophan and its metabolites and hormone crosstalk.

### 4.6 Mechanisms by which tryptophan and its metabolites regulate plant health by inducing defense gene expression

PRs are considered a core family of defense proteins produced by plants during pathogen attack. Among them, *PR-1* has long been regarded as a classical marker gene for SA-mediated disease resistance (Zhang et al., 2022b; Breen et al., 2017). However, recent studies have suggested that tryptophan and its metabolites (especially IAA and melatonin) may directly or indirectly regulate *PR* gene expression through various mechanisms to prevent disease occurrence (Kim et al., 2015). Firstly, transcriptional regulation is the most direct molecular-level by which tryptophan metabolites regulate the expression of defense genes. Zhang et al. (2020) revealed that the novel target of IAA signaling is the resistance gene *PRH1*, which is located in the downstream of *ARF7* and *LBD* and is involved in regulating lateral root development. This discovery connects IAA to the expression network of defense genes and coordinates the outputs of growth and defense. Liu et al. (2024) further confirmed that moderate IAA application can upregulate *PR* gene expression, and IAA and SA pathway defense genes are synergistically regulated to jointly respond to the balance between immune response and plant growth. In addition to IAA, the melatonin biosynthetic enzyme MeASMT2 physically interacts with *PR1* to promote antimicrobial activity and improve the immune response (Guo et al., 2021). Then, the spatial distribution of signaling molecules is closely related to their function, and transporters are critical in regulating the local concentration of tryptophan metabolites and their functions in defense gene expression. The results of tryptophan and ABA homeostasis may reveal the allocation of tryptophan between chloroplasts (synthesis sites) and the cytoplasm (signal functional sites) may affect the supply of downstream metabolites, and indirectly regulate the expression of defense gene (Liu et al., 2022b). Meanwhile, the distribution of melatonin and its synthetic precursors in different subcellular compartments may also determine the efficiency with which it induces defense gene expression. However, the identification of melatonin-specific transporters remains lacking. Meanwhile, the MAPK cascade is a typical pathway through which tryptophan metabolites induce defense gene expression. Lee and Back (2016) clarified a complete signaling cascade, from melatonin receptor sensing to MAPK cascade activation for defense gene transcription. The MAPK cascade (such as MAPKKK3-MKK4/5-MPK3/6) is the core module of plant immune signaling. Its activation may phosphorylate downstream transcription factors (such as WRKY, ERF, etc.), thereby triggering defense gene expression programs. Tryptophan metabolites (particularly IAA and melatonin) regulate ROS/RNS homeostasis, and ROS itself is a potent activator of the MAPK cascade, potentially forming an integrated signal chain of tryptophan metabolites-ROS/RNS-MAPK-defense genes, which constitutes a feedback loop for rapid response to pathogen infection (Mansoor et al., 2024). Overall, tryptophan metabolic enzymes may directly participate in the functional regulation of defense proteins, while IAA and melatonin induce the expression of *PR* gene and activate the MAPK cascade, and crosstalk with SA and ROS/RNS to regulate immune–growth balance through defense genes. However, knowledge gaps remain regarding whether tryptophan and its metabolites directly activate *PR* genes, epigenetic mechanisms, concentration-dependent effects-, and how spatial distribution affects defense gene expression, which require further analysis.

In summary, tryptophan and its metabolites mainly regulate plant health through direct antimicrobial activity, microbiome remodeling, cell wall strengthening, ROS/RNS homeostasis, plant hormone crosstalk, defense-gene expression, and systemic immune activation (**Figure 3**, **Table 1**). Wei et al. (2024) reported that both StS6K2 and StWRKY59 enhance plant resistance to *Phytophthora infestans* through ROS bursts and callose deposition, and participate in secondary metabolite synthesis, plant hormone signal transduction, and plant-pathogen interaction. The same is true for tryptophan and its metabolites. 5-Methoxytryptamine and 5-methoxyindole have been shown to induce defensive responses by inducing H_2_O_2_ and NO production, SA synthesis genes, and *PR* gene expression (Kong et al., 2021). Melatonin has been reported to increase resistance to bacterial infection by activating *PR* genes and SA and ET defense genes (Lee et al., 2014; Kong et al., 2023). It also reduces pathogen damage by inducing JA signaling, ROS homeostasis, and regulating related gene expression (Liu et al., 2022c; Li et al., 2022b). Therefore, tryptophan and its metabolites can act through a single mechanistic pathway and also form a collaborative defense regulatory network through multiple crosstalk and feedback-regulation modules to maintain plant health. Therefore, future research should shift from single mechanisms to network regulation and from controlled experiments to natural ecological settings, in order to truly realize the application potential of tryptophan metabolic pathways in sustainable agriculture.

**Table 1:** Inhibitory effects of tryptophan and its derivatives against pathogens.

Metabolite category	Disease/pathogen	Plant species	Defense mechanism category	Mode of action	Experimental methods	References
Tryptophan	*Fusarium wilt*	Cucumber	Microbiome recruitment by root exudates	Indirect defense	Root tryptophan promotes *B. amyloliquefaciens* colonization to inhibit pathogen	Liu et al., 2017
	Spotted wilt	Tomato	Altered root exudate composition	Indirect defense	Companion planting changes exudate profile (tryptophan) inhibits disease	Zhou et al., 2023
	Leaf spot	Sorghum	Allelopathy /metabolic synergy	Indirect defense	Sorghum-weed mutualism enhances defense through tryptophan accumulation	Li J. Y. et al., 2023
	Stalk rot (*Fusarium proliferatum*)	Maize	Pathogen-induced metabolic reprogramming	Direct/indirect defense	Fungal infection triggers tryptophan accumulation response	Sun et al., 2024
Tryptophan Metabolism overall	Anthracnose	Arabidopsis /crops	Metabolic pathway activation	Multiple mechanisms	Tryptophan metabolism induced by pathogen infection enhances invasive growth resistance	Hiruma et al., 2013
	Sheath rot	Rice	Multi-metabolic pathway synergy	Multiple mechanisms	Pathogen infection upregulates tryptophan metabolism for coordinated defense	Chen et al., 2023
	Black spot (*Alternaria brassicicola*)	Arabidopsis	Nutrient-metabolism cross-regulation	Indirect defense	Nitrogen nutrition regulates resistance by altering tryptophan metabolism	Barrit et al., 2024
Tryptophan synthesis regulation	Cereal cyst nematode	Wheat /Gramineae	Phenylpropanoid metabolism regulation	Indirect defense	TDC confers resistance by affecting phenylalanine ammonia-lyase activity	Zhang et al., 2021c
Auxin (IAA)	Entomopathogenic fungus (*Metarhizium robertsii*)	Crops	Auxin-fungal interaction regulation	Indirect defense	IAA promotes plant growth, regulates fungal pathogens, and reduces disease incidence	Liao et al., 2017
	Anthracnose	Apple	Auxin response gene overexpression	Direct defense	MdIAA24 overexpression increases defense response against *Colletotrichum* infection	Wang et al., 2024
IAA-related metabolites	Fusarium graminearum	Gramineae	Pathogen secondary metabolism inhibition	Direct antibacterial	IAN inhibits 15-ADON toxin production	Luo et al., 2016
	*Phytophthora sojae*	Soybean	Cultivar-specific metabolic regulation	Direct defense	Resistant cultivar RpsJS gene regulates metabolites to enhance defense	Zhu et al., 2018
	Root rot (*Heterobasidion parviporum*)	Conifer	Dark septate endophyte (DSE)-mediated	Indirect defense	Tryptophan-dependent indole compounds produced by DSE enhance disease resistance by promoting nutrient acquisition	Wang et al., 2022b
Tryptamine /5-hydroxy tryptamine (serotonin)	Fungal pathogens (broad-spectrum)	Gramineae	Pathogen-induced accumulation	Direct antibacterial damage repair	Pathogen infection increases tryptamine and serotonin content in leaves	Ishihara et al., 2008, Ishihara et al., 2017
	Fusarium crown rot	Gramineae	Biosynthetic gene overexpression	Direct antibacterial	Infection causes overexpression of tryptophan biosynthetic genes, inducing tryptamine and serotonin levels	Powell et al., 2016
	Citrus canker (Xanthomonas)	Citrus	Metabolite accumulation	Direct antibacterial	Pathogen activates tryptophan metabolism N-methylated tryptamine derivatives accumulate to inhibit pathogen	Saldanha et al., 2022
	Fusarium graminearum	Gramineae	Direct antibacterial activity	Direct antibacterial	5-hydroxytryptamine has direct antibacterial activity against *Fusarium graminearum*	Pasquet et al., 2014
Melatonin	Bacterial stripe pathogen, *Phytophthora nicotianae*	Tobacco	Virulence gene suppression	Direct antibacterial	Melatonin treatment downregulates expression of pathogen virulence-related genes	Zeng et al., 2022
	Cassava common mosaic virus (CsCMV)	Cassava	Synthesis pathway activation	Direct defense	Viral infection transcriptionally activates positively regulating resistance	Wei et al., 2023
Indole glucosinolates (IGs)	Broad-spectrum pathogens	Arabidopsis	CYP-mediated accumulation	Indirect defense	CYP82C2 mutation elevated IGs levels broad-spectrum resistance	Liu et al., 2010
	Black spot (*Alternaria brassicicola*)	Arabidopsis	Specific IGs metabolite accumulation	Direct /Indirect defense	IGs metabolic pathway produces 4MI3G to combat pathogen	Tao et al., 2022; Bednarek et al., 2011
	*P. cucumerina*	Arabidopsis	Myrosinase activation	Direct defense	PEN2 (leaf myrosinase) activates IGs in response to pathogen	Frerigmann et al., 2016
	Broad-spectrum pathogens	Arabidopsis	Auxin homeostasis coupling	Indirect defense	IGs participate in maintaining auxin homeostasis to regulate disease and pest resistance	Malka and Cheng, 2017; Ye et al., 2019
	Broad-spectrum pathogens	Brassicaceae	Hydrolysis product bacteriostasis	Direct antibacterial	Pathogen infection triggers enzymatic hydrolysis of IGs to release thiocyanates, nitriles, and other antimicrobial compounds	Chen et al., 2020; Coca-Ruiz et al., 2024
Camalexin	Verticillium wilt (Verticillium)	Arabidopsis	Pathogen-induced accumulation	Direct antibacterial	Camalexin accumulates in infected roots, inhibiting *Verticillium* growth *in vitro*	Iven et al., 2012; Wu et al., 2022
	Broad-spectrum pathogens	Arabidopsis	MAPK cascade induction	Indirect defense	MAPK cascade induces phytoalexin synthesis to resist pathogens	Ren et al., 2008
	White blister rust (Albugo)	Arabidopsis	Transcriptome reprogramming	Direct defense	Albugo upregulates tryptophan-derived antimicrobial metabolite-related genes, resulting in higher phytoalexin accumulation	Prince et al., 2017
	*A. brassicicola*	Arabidopsis	Transporter-mediated secretion	Direct defense	AtABCG34 mediates camalexin secretion to leaf surface to prevent infection	Khare et al., 2017
	Botrytis cinerea, Pst DC3000, Phytophthora	Arabidopsis	Beneficial bacteria ISR	Indirect defense	Bacillus cereus AR156 triggers ISR by initiating camalexin synthesis	Li et al., 2023a

**Figure 3:**
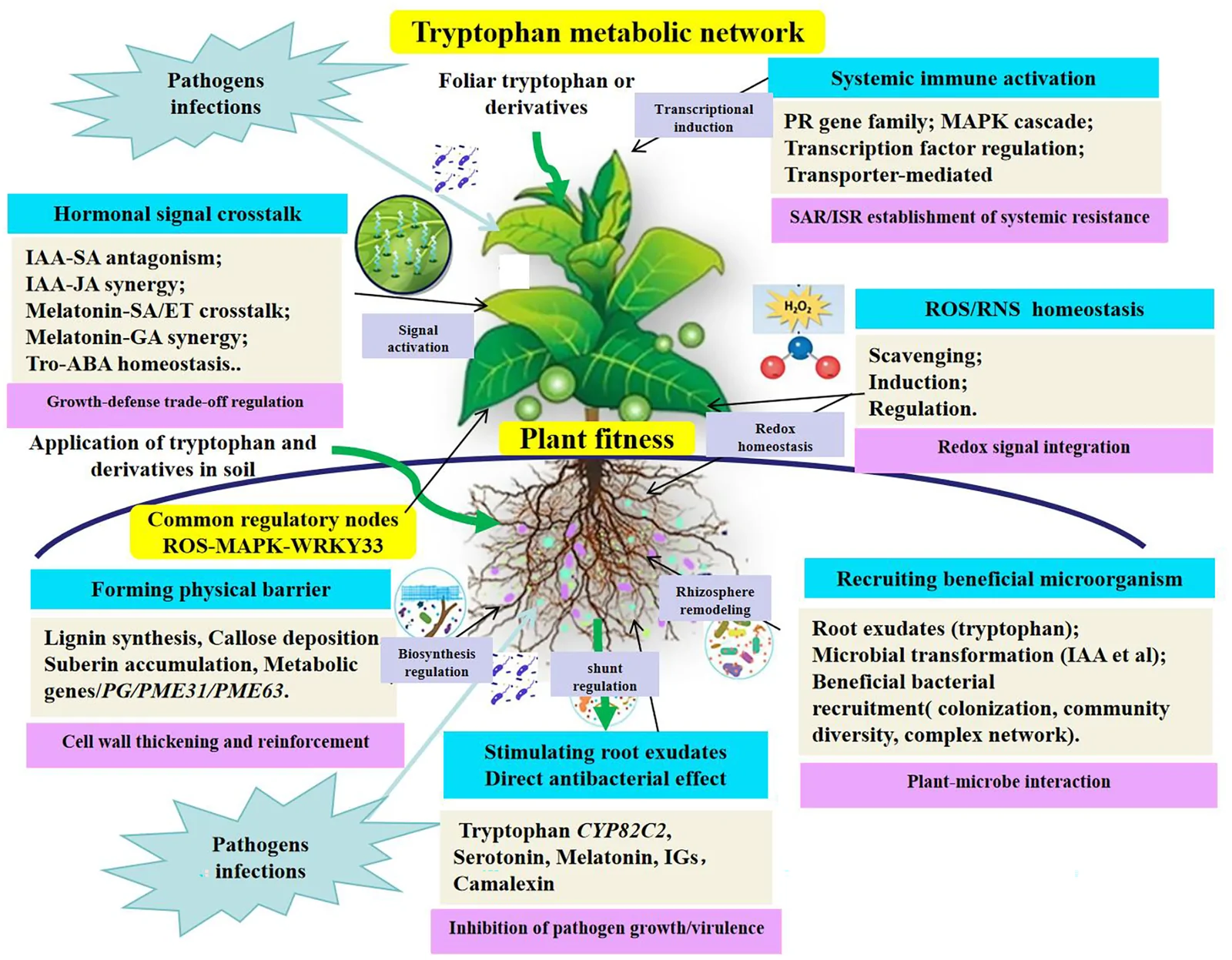
A proposed multi-layered regulatory network of tryptophan and its metabolites in sustaining plant health.

## 5 Application prospects and perspectives of tryptophan and its metabolites

The multiple functions of tryptophan and its metabolites in regulating plant health have been widely confirmed. A multi-level defense network is constituted by direct antimicrobial activity, cell wall strengthening, hormone-signal crosstalk, systemic immune activation, and microbiome remodeling, which helps plants resist pathogen invasion. It is of great significance for elucidating plant disease resistance mechanisms. At the same time, these mechanisms provide rich theoretical resources for the development of new green pesticides, biostimulants, and disease-resistant breeding strategies.

The tryptophan metabolic pathway can produce various physiologically active substances, such as IAA, serotonin, pigments, niacin, coenzymes, and melatonin. It has long been noted that the direct application of tryptophan can promote crop growth, which is mainly relied on IAA produced from tryptophan either in soil or plants (Frankenberger et al., 1990; Arshad et al., 1995). The cyclic peptide of tryptophan possesses antibacterial activity against plant pathogens, which might lay the foundation for the development of new plant protection agents (Camó et al., 2017). Simultaneously, melatonin may serve as an antioxidant, and regulate crop biosynthetic genes through plant hormone crosstalk and signaling molecules, which helps to increase yield and reduce pesticide and fungicide usage while protecting the environment (Yang et al., 2022). To date, 20 melatonin derivatives have been obtained, among which the fungicidal activity of tert-butoxycarbonyl melatonin might increase 25-fold, proving the feasibility of chemical modification of melatonin (Li et al., 2022a). Accordingly, further research on the effects of tryptophan and its metabolites or their bioactive substances on plants may provide valuable assistance to plant biologists and agricultural scientists in different fields, and bring new prospects for the development of plant protection reagents.

With the advancement of microbial technology, tryptophan and its metabolites can attract beneficial microorganisms and reshape the rhizosphere microbial communities, forming a highly stable network with beneficial microorganisms as the core populations, thereby resisting the invasion of pathogen (Rolfe et al., 2019); Wolinska et al., 2021). For example, it has been found that the application of tryptophan and its metabolites promote the rhizosphere colonization of beneficial bacteria such as *Burkholderia* and *Bacillus amyloliquefaciens* and enhance plant growth (Idris et al., 2007; Naveed et al., 2014). However, the combined application of tryptophan and plant growth-promoting rhizobacteria (PGPR) is more conducive to crop health and physiological functions. This might be attributed to the fact that tryptophan and its metabolites are effective resources for enhancing the potential of PGPR (Hassan and Bano, 2015 and tryptophan possesses growth-promoting activity for PGPR (Kravchenko et al., 2004). The combined application of tryptophan and its metabolites with beneficial microorganisms provides novel insights into the cultivation of resistant varieties and may offer new biocontrol agents, possibly offering a more practical biological control strategy.

The application of tryptophan and its metabolites in agriculture is being promoted. Studies have found that there is a synergistic effect between chitosan oligomers and amino acids in inhibiting plant fungal diseases (Buzón-Durán et al., 2021). The binding of melatonin to selenium (Se) induces the activation of antioxidant enzymes and increases the expression of *PR* gene, which enhances plant resistance to pathogenic fungus *Botrytis cinerea* (Zang et al., 2022). Furthermore, they may be used as nanoreactors to induce cascade reactions to produce ROS and RNS, which has great potential for plant health. Thus, further explorations of the combined applicability of tryptophan and its metabolites with other measures and the study of their conjugated complexes have sufficient prospects for the application of environmentally friendly fungicides for plant protection. Simultaneously, we should use scientifically sound application methods and dosages of tryptophan and its metabolites, give full play to their greatest advantages, improve the efficiency of agricultural production and achieve sustainable development.

The tryptophan metabolic network serves as a chemical reservoir and regulatory center in environmental protection plant protection, and holds great potential. However, its practical application relies on resolving a series of interconnected challenges: (i) precision dosing and formulation stability, (ii) edaphic and microbiome-dependent efficacy, (iii) the delicate balance between growth and immunity, (iv) robust multi-site field validation, (v) economic feasibility, and (vi) comprehensive off-target safety assessments. To bridge these translational gaps, it is necessary to consciously combine basic mechanism research with applied agronomic engineering. Following this integrated path, it is crucial for transforming the tryptophan metabolic system from a theoretical resource into a core pillar of sustainable agriculture that can be implemented.

## Data Availability

The original contributions presented in the study are included in the article/supplementary material. Further inquiries can be directed to the corresponding author.
